# Validation of the Turkish version of the Celiac Disease Questionnaire (CDQ)

**DOI:** 10.1186/s12955-015-0272-y

**Published:** 2015-06-19

**Authors:** Ayşegül Aksan, Seyit Mehmet Mercanlıgil, Winfried Häuser, Eda Karaismailoğlu

**Affiliations:** Department of Nutrition and Dietetics, Hacettepe University Faculty of Health Sciences, 06100 Ankara, Turkey; Department of Nutrition and Dietetics, Eastern Mediterranean University Faculty of Health Sciences, via Mersin 10, Famagusta, T.R. North Cyprus Turkey; Department of Psychosomatic Medicine and Psychotherapy, Technische Universität München, D-81865 Munich, Germany; Biostatistics Department, Hacettepe University Faculty of Medicine , 06100 Ankara, Turkey

**Keywords:** Celiac disease, Health related quality of life, Celiac Disease Questionnaire CDQ, Validation, Turkey

## Abstract

**Introduction:**

The aim of the study was to translate, adapt and validate the Celiac Disease Questionnaire (CDQ), which was developed in Germany, for use in Turkey.

**Methods:**

The questionnaire was translated by a forward-backward translation method. Total CDQ score and four subscores (emotional state, gastrointestinal symptoms, worries, social problems) were calculated and their reliability assessed by internal consistency. Reliability of scales was evaluated by internal consistency. Test-retest reliability was assessed by means of a retest after 15 days. Face validity was assessed by patient volunteers and physicians and construct validity was assessed by means of confirmatory factor analysis. Convergent validity was determined by comparing responses to the CDQ with similar subscale scores of the Short Form-36 (SF-36) health survey. Discriminative concurrent criterion validity was determined by comparing the CDQ scores of patients with celiac disease (CD) on a gluten-free diet (GFD) with CD patients not on a GFD.

**Results:**

The Turkish version of the CDQ was completed by 205 patients with celiac disease (Female 146, mean age 31.1 year, ± 10.61). The Cronbach-α coefficent of the subscores ranged between 0.73 and 0.93. Test-retest reliability was 0.99 for all subscores. 42 patients with CD and five gastroenterologists assessed the items of the CDQ to be comprehensible and relevant to the health related quality of life (HRQOL) of CD patients. The confirmatory factor analysis demonstrated acceptable fit indices for the original four subscales of the CDQ. The correlations between the scales of the CDQ and the instrument for validation covering similar dimensions of the HRQOL ranged between 0.61 and 0.93. In all subscores and in the total score, patients not on a GFD showed a significantly reduced HRQOL (all *p* < 0.05) compared to patients on a GFD.

**Conclusion:**

The Turkish version of the CDQ proved to be a reliable and valid instrument for measuring HRQOL in Turkish patients with celiac disease.

**Electronic supplementary material:**

The online version of this article (doi:10.1186/s12955-015-0272-y) contains supplementary material, which is available to authorized users.

## Introduction

Celiac disease (CD), is a prevalent chronic autoimmune disorder that is triggered by the ingestion of wheat gluten and related proteins of rye and barley in genetically susceptible individuals [1–4]. According to the results of different recently-published studies, CD affects between 1:100 and 1:200 individuals in the general population of Turkey [5–7]. Currently the cornerstone of the treatment is a lifelong strict gluten-free diet (GFD) [8], under which gluten is entirely eliminated from food intake and medications. Adherence to a GFD has a positive effect in general on the medical condition of CD patients [1] and, following introduction of the GFD, most patients with CD show improvement not only in terms of gastrointestinal symptoms but also with regards to their psychological well-being. Study results imply that before starting the diet, even CD patients classified as asymptomatic may in fact have had mild symptoms of which they became aware only after having being prescribed a gluten-free diet [9]. Compliance with the diet has been found to be a factor associated with health related quality of life (HRQOL) [10–12], and with improved quality of life in the majority of patients with CD [4, 13, 14].

In recent years, there has been increased medical interest in patients’ subjective perception of the impact chronic disorders have on their lifestyle and general state of health. These factors have therefore become important criteria when assessing the effectiveness of therapeutic intervention [15, 16]. Two basic approaches have been developed for the measurement of quality of life: generic instruments that provide a summary of HRQOL; and specific instruments that focus on problems associated with particular disease states, patient groups, or areas of function [16]. Generic instruments do not assess specific aspects of a certain disease such as the possible social and financial restrictions associated with adherence to a GFD in CD, and are considered to be less sensitive to changes in the patients’ health state [17]. Therefore, the use of disease-specific HRQOL instruments is necessary for clinical studies in gastroenterology [17, 18]. The Celiac Disease Questionnaire (CDQ) is a disease–specific HRQOL questionnaire developed and validated for adult CD patients [17]. Good psychometric properties have been demonstrated for both the German [17] and the Italian version [19].

The purpose of the present study was to translate and validate the CDQ [17] for use in Turkey.

## Methods

### Translation

Two bilingual medical doctors and two interpreters worked on the translation of the CDQ into Turkish. The English version was used as the basis for translation. Translation was carried out by the forward-backward method: First, one bilingual doctor translated the questionnaire from English into Turkish. Subsequently, it was translated back into English by the other bilingual doctor. Finally, these two doctors met together with two interpreters and a doctor with expertise in the field of celiac disease to compare the two English versions and the Turkish translation, and to reconcile discrepancies in order to create one single version.

The abbreviation “CDQ” was kept for the Turkish version for ease of international communication. The Turkish translation of the CDQ is available from the corresponding author on request.

### Patients

Patient members of the Ankara Celiac Association were recruited into the study. Inclusion criteria were: age >19 and <65 years, CD diagnosis based on duodenal biopsy showing a certain degree of villous atrophy and positive endomysial antibodies; the ability to read and understand Turkish; no additional health problems; no vitamin or trace element supplementation within the last year; no tobacco or regular alcohol consumption. The members of Ankara Celiac Association were informed about the study and its inclusion criteria, and of those who had volunteered for study participation, 250 randomly-chosen patients were contacted by telephone or e-mail with a request to complete the CDQ. A total of 239 CDQs were returned. Two weeks after first completing the CDQ, a hundred of the same patients were asked to complete it for a second time. In this second round, 81 CDQs were returned. In addition, 25 randomly-selected bilingual patients were requested to fill out the English version of the CDQ to assess whether the meaning was clear, and whether it was also the same in both languages (21 of the 25 CDQs were returned). The 81 patients who had completed the CDQ a second time were contacted after a further two weeks and asked to fill out the Short Form-36 (SF-36), a similar HRQOL questionnaire. All of those contacted returned the SF-36. Clinical and sociodemographic data of all study participants were collected in order to prevent misinterpretation of results. All patients included in the study had given their prior voluntary informed consent after full written explanation of the aims of the study, including considerations regarding ethics and data protection.

### Psychometric evaluation

Psychometric criteria determined by the IQOLA (International Quality of Life Assessment) project [20] were used to assess the psychometric properties of the Turkish version of the CDQ. Evaluation of data quality involved the floor and ceiling effect (i.e. the percentage of patients scoring the highest or lowest possible score, where the lowest possible percentage is considered an acceptable criterion) and completeness of data (i.e. percentage of items and scales completed, where 90–95 % is considered an acceptable criterion). Scaling assumptions involved item internal consistency (i.e. percentage of items with a Pearson item-scale correlation ≥40 %, criterion being 100 %), discriminant validity (i.e. percentage of items which have a higher Pearson correlation with other scales than with their own scale, criterion is 0 %), internal consistency reliability (Cronbach’s alpha coefficient, criterion is >0.7) and test-retest reliability (Pearson correlation, criterion is >0.7).

### Questionnaires

The CDQ is an instrument consisting of 28 items divided into four subscales : emotion (seven items), gastrointestinal symptoms (seven items), worries (seven items), social problems (seven items). The emotional states include depressed, relaxed, happy, restless, physically fatigued and tearful; gastrointestinal symptoms include abdominal cramps, bloating, incomplete bowel evacuation, loose bowels, aberrant bowel movement, belching and nausea; worries include fear of medical examinations, health insurance issues, fear of cancer, fears concerning inheritance to children, gluten-free diet costs and concerns associated with living a gluten-free life; social problems include lack of social understanding, exclusion from others, family/friends, sports, a professional career, social or meal invitations and sexual activities [17, 19].

Each subscale is scored using a seven point Likert scale in which “7” corresponds to the best state and “1” to the worst, with 2 of the items requiring reverse coding. The scores of each area range from 0 to 49 and the total CDQ score ranges from 0 to 196 [17].

The sociodemographic questionnaire includes demographic questions to assess gender, marital status, age, lifestyle variables (regular cigarette smoking, regular alcohol intake, regular participation in sport), present occupational status and educational status.

The medical questionnaire includes questions to assess medical history of the disease, adherence to a gluten-free diet, body mass index, prior or current celiac-associated disease, other diseases and current medication.

The Medical Outcome Study Short Form-36 (SF-36) [21] was used for validation. SF-36 has been shown to be a reliable and valid instrument in Turkish patients [22], and consists of 36 items with eight subscales: physical functioning (ten items), social functioning (two items), role limitations due to physical problems (four items), role limitations due to emotional problems (three items), mental health (five items), energy and vitality (four items), pain (two items), and general perception of health (five items). For each variable item, scores are coded, added and transformed onto a scale from 0 (worst possible health state measured by the questionnaire) to 100 (best possible health state) [21, 22].

### Validation methods and hypothesis

The methods used to validate the CDQ were as follows:

- Acceptance, assessed by the proportion of missing or invalid items.
- Reliability of the scales, assessed by internal consistency as determined by Cronbach’s α coefficient, which measures the overall correlation between items within a scale. A correlation level of 0,7 and higher is considered desirable [23].
- Test-retest reliability was assessed in 81 patients who completed the CDQ twice within 15 days.
- Face validity in CD patients had been assessed in German subjects for the original questionnaire [17]. In this research, face validity of the Turkish version was evaluated by 42 patients with CD and five physicians. We tested face validity by asking the patients and physicians to evaluate whether the CDQ subsections were relevant or irrelevant to CD.
- Factorial/construct validity was assessed by confirmatory factor analysis, which was used to confirm the factors or sub-scales determined in the original scale. Normed fit index (NFI), comparative fit index (CFI) and chi-squared statistics were used for evaluating model fit.
- Convergent validity was determined by comparing responses to the CDQ with similar subscale scores of the SF-36, which has been shown to be a reliable and valid instrument in Turkish patients. The convergent validity was fulfilled when the scale scores for related concepts showed moderate to high correlation (Spearman rank correlation coefficients >0,4) [23]. The expectation is of a higher correlation in similar dimensions (shown bold in Table 3) and a lower correlation in dissimilar dimensions, indications that the CDQ has clear outcomes.
- Discriminant concurrent criterion validity was determined by comparing the CDQ scores of patients with CD on a GFD with those patients not on a GFD. We predicted that patients on a GFD would show increased HRQOL scores compared with patients not on a GFD.

### Statistical analysis

The Statistical Package for Social Sciences (SPSS 21.0 package for Windows) was used to analyze the data.

Descriptive statistics were applied to report continuous variables as means ± standard deviation (SD). Categorical data were given as counts and percentages. Chi-square and Analysis of Variance (ANOVA) were used for analysis of categorical and continuous data, respectively, while the correlation between CDQ and other dependent variables were described by Pearson and Spearman correlation tests. Pearson correlation analysis was calculated for normally-distrubuted and Spearman correlation for abnormally-distributed variables.

Confirmatory factor analysis was executed to determine factorial/construct validity. NFI (>0.90 acceptable fit), CFI (>0.90 acceptable fit), Root Mean Square Residual (<0.08 acceptable fit) were calculated and chi-squared statistics were used for evaluating model fit [24].

Cronbach’s alpha was computed in order to assess internal consistency and test–retest reliability, respectively. Discriminant validity was evaluated by Mann Whitney rank-sum test. All tests were two-tailed with the significance level set at *p* < 0.05.

The study was approved by Hacettepe Üniversitesi Girişimsel Olmayan Klinik Araştırmalar Etik Kurulu with the reference number HEK 12/113–24.

## Results

### Patients

Twenty-one patients were excluded due to the exclusion criteria of the study (12 patients due to tobacco consumption, 9 patients due to additional health problems), while thirteen patients originally included in the study could not be contacted or were considered to be lost to follow-up and eleven patients never returned the questionnaire. At study completion, two hundred and five (146 females, 59 males, mean age 31.1 years ± 10.61) patients with CD were included. Sociodemographic and medical data of the patients are presented in Table 1.

**Table 1:** Sociodemographic and medical data of patients (*n* = 205)

Variable	*N*	%
Sex		
Female	146	71.2
Male	59	28.8
Age(years) (mean ± standard deviation)	32.3 ± 9.09	
Age at diagnosis		
5–10	19	9.3
11–20	50	24.4
21–30	78	38.0
31–40	41	20.0
41–50	16	7.8
>50	1	0.5
Educational status		
Illiterate	1	0.5
Literate	5	2.4
Primary school graduate	8	3.9
Middle school graduate	17	8.3
High school graduate	71	34.6
College graduate	103	50.2
Compliance to gluten-free diet		
All of the time	143	69.8
A good bit of the time	44	21.5
Some of the time	15	7.3
Hardly any of the time	2	1.0
None of the time	1	0.5
Income status		
<1000 TL^a^	46	22.4
1000–2000 TL^a^	69	33.7
2000–3000 TL^a^	39	19.0
3000–4000 TL^a^	26	12.7
4000–5000 TL^a^	7	3.4
>5000 TL^a^	18	8.8

^a^TL means Turkish Liras

### Psychometric evaluation

Floor and ceiling effect was found to be 0 % for the total score, 1 % for the emotional and social subscale scores, and 0 % for the worries and gastrointestinal symptoms subscale scores. Completeness of data was 100 %, item internal consistency was 100 %, discriminant validity was 0 %, minimum internal consistency reliability was 0.73, and minimum test-retest reliability was 0.99. Based on these evaluations, the psychometric properties of the Turkish version of the CDQ were found to be acceptable.

### Reliability

Data on the internal consistency of the scales are presented in Table 2. Cronbach’s α ranged between 0.73 and 0.93, and test-retest reliability was 0.99 for all subscales.

**Table 2:** The Internal consistency and test-retest reliability of CDQ subscales (*n* = 81)

CDQ subscale	Mean (standard deviation)	Cronbach’s alpha	Spearman correlation coefficient (retest reliability)
Emotion	28.6(9.0)	0.93	0.99
Social	34.0(8.1)	0.75	0.99
Worries	28.0(8.5)	0.73	0.99
Bowel	34.2(8.3)	0.86	0.99
Total	124.8(28.1)		0.99

### Validity

Face validity; 42 volunteers recruited from the participating patients with CD and all physicians assessed the items of the CDQ to be comprehensible, and indicated that the items were relevant to HRQOL for CD patients. The patients and the doctors agreed that the listed CDQ items are relevant issues for individuals with celiac disease.

Construct validity; The subscale characteristics are presented in Table 3. There were no significant differences in the CDQ subscales or in the total score between males and females or between patients of different educational backgrounds. Furthermore, there were no significant correlations with age (Table 4).

**Table 3:** Correlations of the CDQ with SF 36 subscales (Spearman Correlation)

CDQ Items					
	Emotion	Social	Worries	Gastrointestinal	Total
SF-36 Items					
Physical function	0.52 (<0.001)	0.49(<0.001)	0.40(0.001)	**0.68(<0.001)**	0.67(<0.001)
Role-physical	0.50(<0.001)	0.45(<0.001)	0.38(0.002)	**0.61(<0.001)**	0.62(<0.001)
Bodily pain	0.52(<0.001)	0.37(0.003)	0.24(0.055)	**0.85(<0.001)**	0.57(<0.001)
General health perception	0.54(<0.001)	0.55(<0.001)	0.56(<0.001)	**0.69(<0.001)**	0.73(<0.001)
Vitality	**0.86(<0.001)**	0.52(<0.001)	0.31(0.014)	0.61(<0.001)	0.72(<0.001)
Social functioning	0.59(<0.001)	**0.88(<0.001)**	0.47(<0.001)	0.46(<0.001)	0.76(<0.001)
Role-emotion	**0.63(<0.001)**	0.25(0.047)	0.17(0.177)	0.33(0.008)	0.42(0.001)
Mental health	**0.93(<0.001)**	0.57(<0.001)	0.32(0.009)	0.60(<0.001)	0.75(<0.001)

r = 0.4 to 0.8 expected

The highest rates of correlation for the respective items are shown in bold

**Table 4:** Subscores of the Turkish CDQ subcategorized by gender, age and education

	Emotion		Social		Worries		Gastro-intestinal		Total	
	Mean (SD)	*p*	Mean (SD)	*p*	Mean (SD)	*p*	Mean (SD)	*p*	Mean (SD)	*p*
Gender										
Male (*n* = 59)	28.58 (9.30)	0.891	32.55 (8.10)	0.115	27.24 (9.34)	0.291	34.97 (8.00)	0.540	123.34 (29.86)	0.512
Female (*n* = 146)	28.55 (8.95)		34.56 (8.03)		28.32 (8.13)		33.89 (8.37)		125.32 (27.37)	
Age										
19–40 (*n* = 161)	28.01 (9.12)	0.093	33.46 (8.10)	0.065	27.63 (8.82)	0.187	34.06 (8.48)	0.777	123.17 (28.58)	0.131
41–64 (*n* = 44)	30.55 (8.51)		35.91 (7.81)		29.39 (7.06)		34.70 (7.47)		130.55 (25.51)	
Educational Level										
Illiterate (*n* = 1)	44 (−)	0.213	45 (−)	0.603	38 (−)	0.258	47 (−)	0.071	174 (−)	0.259
Literate (*n* = 5)	32.80 (11.09)		36.00 (8.75)		34.20 (9.96)		40.60 (3.91)		143.60 (21.55)	
Primary school graduate (*n* = 8)	27.38 (9.07)		33.13 (11.83)		22.75 (9.42)		33.75 (7.42)		117.00 (34.33)	
Middle school graduate (*n* = 17)	32.65 (11.48)		33.59 (7.12)		25.59 (10.82)		37.12 (9.47)		128.94 (35.18)	
High school graduate (*n* = 71)	27.86 (8.96)		33.21 (8.00)		28.38 (8.08)		33.85 (8.68)		123.30 (27.95)	
College graduate (*n* = 103)	28.10 (8.37)		34.45 (8.00)		28.17 (8.05)		33.56 (7.79)		124.27 (26.34)	

Confirmatory factor analysis was performed to verify the construct validity of the scale. As a result of analysis of the model, fit indices were examined (NFI = 0.91, CFI = 0.93, RMR = 0.073). Accordingly, the chi-square value was found to be statistically significant (*χ*2 = 794.79, *p* <0.001, *χ*2/dfa: 794.79/344 = 2,31). Fit indices accepted < 5 for *χ*2/dfa, >0.90 for NFI and CFI, <0.08 for RMR [23].

Convergent validity; The correlations (Spearman rank-order correlation) between the scales of the CDQ and the instrument for validation covering similar dimensions of the HRQOL ranged between 0.61 and 0.93 (Table 3). High scores in CDQ and SF-36 indicate a good HRQOL.

Discriminant concurrent criterion validity: Differences in the CDQ subscales between patients on a GFD and those not on a GFD are presented in Table 5. In all subscales and in terms of total score, patients not on a GFD showed a significantly reduced HRQOL (total *p* < 0.05) compared to patients adhering to a GFD.

**Table 5:** Health-related quality of life of CD patients on a GFD compared with those not on a GFD, as measured by the Turkish CDQ

CDQ subscale	GFD (n = 143)	Not on GFD (n = 62)	*P*
	Mean (SD)	Mean (SD)	
Emotion	29,71 (9.05)	25,90 (8.48)	0.003
Social	35,54 (7.49)	30,40 (8.30)	<0.001
Worries	28,84 (8.43)	26,10 (8.38)	0.021
Gastrointestinal	35,46 (7.81)	31,29 (8.60)	0.002
Total	129,55 (26.82)	113,69 (27.90)	<0.001

*GFD* Gluten-free diet

## Discussion

The number of individuals diagnosed with celiac disease has increased in recent years [25]. Due to the necessity for lifelong changes in lifestyle in persons with CD, health-related quality of life has become an important focus of attention [17, 26].

The CDQ is an economic, reliable and valid disease-specific instrument for the assessment of HRQOL in patients with CD [17]. In this study, the Turkish adaptation of the CDQ was validated for the first time for patients with CD in living Turkey.

The enrollment rate of approximately 82 % of patients who had expressed interest in study participation is acceptable. A natural sounding, easily comprehensible translation of the CDQ was obtained in order to reduce all possible cultural bias to a minimum. Most sociodemographic parameters of this Turkish sample are similar to those of other celiac health surveys in Germany [17] and Italy [19] but HRQOL scores in all subscales and in total were lower in this Turkish study population than in German [17] and Italian [19] samples. Results of the study show that scale scores were unaffected by the gender, age and educational backgrounds of those completing the questionnaire.

Regarding the construct validity of the CDQ, our study confirmed the factorial structure by confirmatory factor analysis for the first time. The confirmatory factor analysis demonstrated the homogeneity of the items and also the internal consistency to be satisfactory for the original four dimensions of the CDQ. Furthermore, the Turkish version of CDQ showed good overall psychometric properties with excellent internal consistency for the overall score and each of the four subscores.

The correlations of the Turkish CDQ with the SF-36, an HRQOL scale instrument validated in Turkish, were significant (Spearman correlation 0.61-0.93) and similar to the external validity coefficients of other validation studies of the CDQ [17, 19].

Our analysis also confirmed the ability of the CDQ to discriminate between patients on a gluten-free diet and those not on a gluten-free diet. Due to the discriminant validity of the Turkish questionnaire, the scores of patients on a gluten-free diet were - as expected - observed to be significantly higher than in patients who did not adhere to a gluten-free diet. This criterion was therefore identified as a classifying parameter.

Some limitations of the study must be considered: Within the setting of this study, a test of responsiveness (the ability of a questionnaire to detect clinically important changes over time) was not possible. The patients were recruited from the Celiac Disease Association (Çölyak Derneği) and the selection process offers no guarantee that these patients were representative for individuals with celiac disease in the Turkish population as a whole. Therfore, a potential selection bias in our sample cannot be entirely excluded.

The use of internationally validated instruments is strongly recommended to allow international comparison of the results of national studies. Transcultural comparisons of adult celiac patients can be conducted using the CDQ. There are also validated celiac-specific quality of life instruments available for children and adolescents [27] which should be validated in future.

In conclusion, this study showed the new Turkish version of the CDQ to be a valid and reliable instrument for measuring HRQOL in Turkish patients with celiac disease. The Turkish version of the CDQ including information on copyright and contact information are included in the electronic Additional file 1.

## Additional file

Additional file 1:
**Turkish Version of Celiac Disease Questionnaire (CDQ).**

## Abbreviations

ANOVA: Analysis of Variance
CD: Celiac disease
CDQ: Celiac Disease Questionnaire
CFI: Comparative fit index
DFA: Detrended fluctuation analysis
GFD: Gluten-free diet
HRQOL: Health-related quality of life
IQOLA: International quality of life assessment
NFI: Normed fit index
RMR: Root mean square residual
SF-36: Short form-36 health survey

## References

[1] Green PHR, Jabri B (2003). Coeliac disease. Lancet.

[2] Briani C, Samaroo D, Alaedini A (2008). Celiac disease: from gluten to autoimmunity. Autoimmun Rev.

[3] Lee AR, Ng DL, Diamond B, Ciaccio EJ, Green PH (2012). Living with coeliac disease: survey results from the U.S.A. J Hum Nutr Diet.

[4] Nachman F, Mauriño E, Vázquez H, Sfoggia C, Gonzalez A, Gonzalez V (2009). Quality of life in celiac disease patients: prospective analysis on the importance of clinical severity at diagnosis and the impact of treatment. Dig Liver Dis.

[5] Gursoy S, Guven K, Simsek T, Yurci A, Torun E, Koc N (2005). The prevalence of unrecognized adult celiac disease in Central Anatolia. J Clin Gastroenterol.

[6] Tatar G, Elsurer R, Simsek H, Balaban YH, Hascelik G, Ozcebe OI (2004). Screening of tissue transglutaminase antibody in healthy blood donors for celiac disease screening in the Turkish population. Dig Dis Sci.

[7] Elsurer R, Tatar G, Simsek H, Balaban YH, Aydinli M, Sokmensuer C (2005). Celiac disease in the Turkish population. Dig Dis Sci.

[8] Garcia-Manzanares A, Lucendo AJ (2011). Nutritional and dietary aspects of celiac disease. Nutr Clin Pract.

[9] Mustalahti K, Lohiniemi S, Collin P, Vuolteenaho N, Laippala P, Maki M (2002). Gluten-free diet and quality of life in patients with screen-detected celiac disease. Eff Clin Pract.

[10] Zarkadas M, Cranney A, Case S, Molloy M, Switzer C, Graham ID (2006). The impact of a gluten-free diet on adults with coeliac disease: results of a national survey. J Hum Nutr Diet.

[11] Usai P, Minerba L, Marini B, Cossu R, Spada S, Carpiniello B (2002). Case control study on health-related quality of life in adult coeliac disease. Dig Liver Dis.

[12] Kurppa K, Collin P, Mäki M, Kaukinen K (2011). Celiac disease and health-related quality of life. Expert Rev Gastroenterol Hepatol.

[13] Nachman F, del Campo MP, González A, Corzo L, Vázquez H, Sfoggia C (2010). Long-term deterioration of quality of life in adult patients with celiac disease is associated with treatment noncompliance. Dig Liver Dis.

[14] Cranney A, Zarkadas M, Graham ID, Butzner JD, Rashid M, Warren R (2007). The Canadian celiac health survey. Dig Dis Sci.

[15] Bech P (1993). Quality of life measurements in chronic disorders. Psychother Psychosom.

[16] Guyatt GH, Feeny DH, Patrick DL (1993). Measuring health-related quality of life. Ann Intern Med.

[17] Häuser W, Gold J, Stallmach A, Caspary WF, Stein J (2007). Development and Validation of the Celiac Disease Questionnaire (CDQ), a disease-specific health-related quality of life measure for adult patients with celiac disease. J Clin Gastroenterol.

[18] Yacavone RF, Locke GR, Provenzale DT, Eisen GM (2001). Quality of life measurement in gastroenterology: what is available?. Am J Gastroenterol.

[19] Marchese A, Klersy C, Biagi F, Balduzzi D, Bianchi PI, Trotta L (2013). Quality of life in coeliac patients: Italian validation of a coeliac questionnaire. Eur J Intern Med.

[20] Ware JE, Gandek B (1998). Methods for testing data quality, scaling assumptions, and reliability: the IQOLA Project approach. J Clin Epidemiol.

[21] Ware JE, Kosinski M (2001). SF-36 physical & mental health summary scales: a manual for users of version 1: Quality Metric Inc.

[22] Kocyigit H, Aydemir O, Olmez N, Memis A (1999). Reliability and validity of the Turkish version of Short-Form-36 (SF-36). Turkish J Drugs Therap.

[23] Fletcher A, Gore S, Jones D, Fitzpatrick R, Spiegelhalter D, Cox D (1992). Quality of life measures in health care. II: Design, analysis, and interpretation. BMJ (Clin Res).

[24] Schermelleh-Engel K, Moosbrugger H, Müller H (2003). Evaluating the fit of structural equation models: tests of significance and descriptive goodness-of-fit measures. Int J Methods Psychiatr Res.

[25] Ludvigsson JF, Rubio-Tapia A, van Dyke CT, Melton LJ, Zinsmeister AR, Lahr BD (2013). Increasing incidence of celiac disease in a North American population. Am J Gastroenterol.

[26] Dorn SD, Hernandez L, Minaya MT, Morris CB, Hu Y, Leserman J (2010). The development and validation of a new coeliac disease quality of life survey (CD-QOL). Aliment Pharmacol Ther.

[27] van Doorn RK, Winkler LM, Zwinderman KH, Mearin ML, Koopman HM (2008). CDDUX: a disease-specific health-related quality-of-life questionnaire for children with celiac disease. J Pediatr Gastroenterol Nutr.

