# Supplementary material for: Validation of the Turkish version of the Celiac Disease Questionnaire (CDQ)
Source: Health Qual Life Outcomes. 2015 Jun 19;13:82. doi: 10.1186/s12955-015-0272-y (PMC4472159; doi:10.1186/s12955-015-0272-y)
Supplement: Additional file 1: — Turkish Version of Celiac Disease Questionnaire (CDQ). [file 12955_2015_272_MOESM1_ESM.pdf]

ÇÖLYAK HASTALARINDA SAĞLIKLA İLİŞKİLİ YAŞAM KALİTESİ (CDQ)

Bu anket son iki haftalık zaman dilimi içerisinde nasıl hissettiğinizi değerlendirmek için geliştirilmiştir. Bu amaçla size çölyak hastalığınız ile ilgili semptomlar, genel iyilik haliniz, kendinizi nasıl hissettiğiniz, ruhsal durumunuz ile ilgili 28 adet soruya cevap vermeniz beklenmektedir. Her bir soru için 1 ve 7 arasında puanlarla değerlendirilecek 7 olası cevap bulunmaktadır. Lütfen son iki haftalık süreçte sizi en iyi tanımlayan cevabı dikkatlice seçerek işaretleyiniz. Eğer cevaplamak istemediğiniz soru, ya da size uygun olmayan bir ifade olursa (örneğin cinsel yaşam ile ilgili bir soru) olursa lütfen boş bırakınız.

1. Geçtiğimiz iki hafta boyunca kaç kez barsak hareketleriniz sebebiyle ani tuvalete girme ihtiyacı hissettiğiniz için günlük yaşamınız kötü etkilendi?

1	2	3	4	5	6	7
Her zaman	Çoğu zaman	Sıklıkla	Arasıra	Nadiren	Neredeyse hiç	Hiçbir zaman

2. Geçtiğimiz iki hafta boyunca ne sıklıkla kendinizi fiziksel olarak yorgun veya bitkin hissettiniz?

1	2	3	4	5	6	7
Her zaman	Çoğu zaman	Sıklıkla	Arasıra	Nadiren	Neredeyse hiç	Hiçbir zaman

3. Geçtiğimiz iki hafta boyunca ne sıklıkla kendinizi bıkkın, tahammülsüz veya huzursuz hissettiniz?

1	2	3	4	5	6	7
Her zaman	Çoğu zaman	Sıklıkla	Arasıra	Nadiren	Neredeyse hiç	Hiçbir zaman

4 Geçtiğimiz iki hafta boyunca kaç defa çölyak hastalığınız nedeniyle bir yakınınız ya da arkadaşınız tarafından yapılan yemek davetini reddettiniz veya davete katılmaktan kaçındınız?

1	2	3	4	5	6	7
Her zaman	Çoğu zaman	Sıklıkla	Arasıra	Nadiren	Neredeyse hiç	Hiçbir zaman

5.Geçtiğimiz iki hafta boyunca ne sıklıkla ishal oldunuz?

1	2	3	4	5	6	7
Her zaman	Çoğu zaman	Sıklıkla	Arasıra	Nadiren	Neredeyse hiç	Hiçbir zaman

6.Geçtiğimiz iki hafta boyunca zihinsel olarak ne kadar enerjiktiniz?

1	2	3	4	5	6	7
Hiç enerjim yoktu	Çok az enerjim vardı	Az enerjim vardı	Biraz enerjim vardı	Ortalama enerjim vardı	Çok enerjim vardı	Enerji doluydum

7. Geçtiğimiz iki hafta boyunca ne sıklıkla çocuklarınızın çölyak hastalığını genetik olarak sizden almış olduğu ya da olabileceği endişesine kapıldınız?

1	2	3	4	5	6	7
Her zaman	Çoğu zaman	Sıklıkla	Arasıra	Nadiren	Neredeyse hiç	Hiçbir zaman

8. Geçtiğimiz iki hafta boyunca ne sıklıkla karnınıza kramp girmesi nedeniyle sorun yaşadınız?

1	2	3	4	5	6	7
Her zaman	Çoğu zaman	Sıklıkla	Arasıra	Nadiren	Neredeyse hiç	Hiçbir zaman

9. Geçtiğimiz iki hafta boyunca çölyak hastalığınız yüzünden günlük aktivitelerinizde veya sportif faaliyetleinizde zorluk yaşadınız mı?

1		Çok fazla zorlandım, herhangi bir aktivitede bulunmam imkansızdı
2		Oldukça fazla zorlandım
3		Fazlaca zorlandım
4		Biraz zorlandım
5		Az zorlandım
6		Neredeyse hiç zorlanmadım
7		Hiç zorlanmadım, çölyak hastalığı günlük aktivitelerimi veya sportif faaliyetlerimi etkilemedi

10. Geçtiğimiz iki hafta boyunca ne sıklıkla kendinizi depresif ve bezgin hissettiniz?

1	2	3	4	5	6	7
Her zaman	Çoğu zaman	Sıklıkla	Arasıra	Nadiren	Neredeyse hiç	Hiçbir zaman

11. Geçtiğimiz iki hafta boyunca ne sıklıkla şişkinlik veya gaz şikayetiniz oldu?

1	2	3	4	5	6	7
Her zaman	Çoğu zaman	Sıklıkla	Arasıra	Nadiren	Neredeyse hiç	Hiçbir zaman

12. Çölyak hastası olan insanlar genellikle hastalıkları ile ilgili korku ve endişe duyarlar. Geçtiğimiz iki hafta boyunca ne kadar sıklıkla çölyak hastalığınız sonucunda kanser olabileceğiniz endişesine kapıldınız veya bundan korktunuz?

1	2	3	4	5	6	7
Her zaman	Çoğu zaman	Sıklıkla	Arasıra	Nadiren	Neredeyse hiç	Hiçbir zaman

13 Geçtiğimiz iki hafta boyunca ne sıklıkla büyük tuvaletinizi tam yapamadığınız hissine kapıldınız?

1	2	3	4	5	6	7
Her zaman	Çoğu zaman	Sıklıkla	Arasıra	Nadiren	Neredeyse hiç	Hiçbir zaman

14. Geçtiğimiz iki hafta boyunca ne sıklıkla kendinizi rahatlamış ve sakin hissettiniz?

7	6	5	4	3	2	1
Her zaman	Çoğu zaman	Sıklıkla	Arasıra	Nadiren	Neredeyse hiç	Hiçbir zaman

15. Geçtiğimiz iki hafta boyunca ne sıklıkla çölyak hastalığınız nedeniyle çevrenizden soyutlandığınızı veya insanlar tarafından dışlandığınızı hissettiniz?

1	2	3	4	5	6	7
Her zaman	Çoğu zaman	Sıklıkla	Arasıra	Nadiren	Neredeyse hiç	Hiçbir zaman

16. Geçtiğimiz iki hafta boyunca ne sıklıkla ağlamaklı ve üzgün hissettiniz?

1	2	3	4	5	6	7
Her zaman	Çoğu zaman	Sıklıkla	Arasıra	Nadiren	Neredeyse hiç	Hiçbir zaman

17. Geçtiğimiz iki hafta boyunca ne sıklıkla tekrarlayan geğirmeye katlanmak zorunda kaldınız?

1	2	3	4	5	6	7
Her zaman	Çoğu zaman	Sıklıkla	Arasıra	Nadiren	Neredeyse hiç	Hiçbir zaman

18. Geçtiğimiz iki hafta boyunca çölyak hastalığı cinsel aktivitenizi ne derecede kısıtladı?

1		Cinsellik yaşamadım
2		Oldukça fazla kısıtlandım
3		Fazlaca kısıtlandım
4		Biraz kısıtlandım
5		Az kısıtlandım
6		Neredeyse hiç kısıtlanmadım
7		Kısıtlanmadım

19. Geçtiğimiz iki hafta boyunca ne sıklıkla öğürme ya da mide bulantısına katlanmak zorunda kaldınız?

1	2	3	4	5	6	7
Her zaman	Çoğu zaman	Sıklıkla	Arasıra	Nadiren	Neredeyse hiç	Hiçbir zaman

20. Geçtiğimiz iki hafta boyunca ne sıklıkla aile üyeleriniz ya da arkadaşlarınız gibi sizin için önemli olan insanların çölyak hastalığınıza karşı yeterince anlayışlı davranmadıklarını hissettiniz?

1	2	3	4	5	6	7
Her zaman	Çoğu zaman	Sıklıkla	Arasıra	Nadiren	Neredeyse hiç	Hiçbir zaman

21. Geçtiğimiz iki hafta boyunca hayatınızdan ne kadar hoşnut, mutlu veya memnundunuz?

1		Oldukça memnuniyetsizdim, çoğu zaman mutsuzdum
2		Memnuniyetsizdim, mutsuzdum
3		Genelllikle memnuniyetsizdim, mutsuzdum
4		Genellikle memnundum, hoşnuttum
5		Çoğunlukla memnundum, mutluydum
6		Çoğunlukla çok memnundum, mutluydum
7		Çok memnundum, daha mutlu veya hoşnut olamazdım

22. Geçtiğimiz iki hafta boyunca ne sıklıkla iş arkadaşlarınızın ya da müdürlerinizin çölyak hastalığınıza karşı yeterince anlayışla davranmadığını hissettiniz?

1	2	3	4	5	6	7
Her zaman	Çoğu zaman	Sıklıkla	Arasıra	Nadiren	Neredeyse hiç	Hiçbir zaman

23. Geçtiğimiz iki hafta boyunca ne sıklıkla çölyak hastalığınızdan dolayı profesyonel yaşamınızda/kariyerinizde kısıtlandığınızı hissettiniz?

1	2	3	4	5	6	7
Her zaman	Çoğu zaman	Sıklıkla	Arasıra	Nadiren	Neredeyse hiç	Hiçbir zaman

24. Geçtiğimiz iki hafta boyunca ne sıklıkla glutensiz ürünlere ulaşma zorluğu size kendinizi sıkıntılı hissettirdi?

1	2	3	4	5	6	7
Her zaman	Çoğu zaman	Sıklıkla	Arasıra	Nadiren	Neredeyse hiç	Hiçbir zaman

25. Geçtiğimiz iki hafta boyunca ne sıklıkla glutensiz ürünlerin masrafı (fiyat, reçete, geri ödeme vb...) ve diğer çölyak hastalığı tedavileri size kendinizi maddi açıdan sıkıntılı hissettirdi?

1	2	3	4	5	6	7
Her zaman	Çoğu zaman	Sıklıkla	Arasıra	Nadiren	Neredeyse hiç	Hiçbir zaman

26. Geçtiğimiz iki hafta boyunca ne sıklıkla çölyak hastalığınız hakkında doktorlarınızdan yeterli yönlendirme alamadığınızı düşündünüz?

1	2	3	4	5	6	7
Her zaman	Çoğu zaman	Sıklıkla	Arasıra	Nadiren	Neredeyse hiç	Hiçbir zaman

27. Geçtiğimiz iki hafta boyunca ne sıklıkla çölyak hastalığınıza çok geç tanı konulduğunu konusunda endişe duydunuz?

1	2	3	4	5	6	7
Her zaman	Çoğu zaman	Sıklıkla	Arasıra	Nadiren	Neredeyse hiç	Hiçbir zaman

28. Geçtiğimiz iki hafta boyunca ne sıklıkla çölyak hastalığınızla ilgili kan alınması ya da endoskopi gibi tıbbi tetkik yaptırma korkusu yaşadınız?

1	2	3	4	5	6	7
Her zaman	Çoğu zaman	Sıklıkla	Arasıra	Nadiren	Neredeyse hiç	Hiçbir zaman

CDQ – Testin Uygulanması ve Yorumlanması

Test Kullanımı

CDQ yetişkinler için geliştirilmiştir (≥ 18 Yaş), testin uygulanmasıyla ilgili talimatı ilk sayfada bulabilirsiniz. Testin uygulanması yaklaşık 10 dakika sürmektedir, anlaşılması kolay sorulardan oluşmakta, sıklıkla anlama güçlüğü çekilmemektedir. Teste başlamadan önce hasta tüm soruları kendisinin yanıtlaması, partnerine ya da ailesine sormaması konusunda bilgilendirilmelildir. Eğer hastanın yanında aile veya arkadaşlar varsa, test dolumunun sırasında hastayı etkileyebilecek konuşmalar oluşmamasına dikkat edilmelidir. Testin bitiminin ardından araştırmacı tüm soruların yanıtlandığını kontrol etmeli, çalışmaya katılan hastanın değerlendirme ile ilgili soruları varsa yanıtlamalıdır.

CDQ Değerlendirilmesi

Hastanın ölçek puanları, ilgili maddelerinin sayılarını alarak elde edilir. Alt ölçeklerin değerleri, CDQ - değerlendirme kağıdında yazılan maddelerin ölçeklerini toplayarak ortaya çıkmaktadır.

Duygu (D)

Soru 2 + 3 + 6 + 10 + 14 + 16 + 21 = D

Sosyal (S)

Soru 4 + 9 + 15 + 18 + 20 + 23 + 23 = S

Endişe (E)

Soru 7 + 12 + 24 + 25 + 26 + 27 + 28 = E

Gastrointestinal (GI)

Soru 1 + 5 + 8 + 11 + 13 + 17 + 19 = GI

Toplam (To)

D +	S +	E +	GI =	To

Test Yorumu

Değerlendirme puanı her soruya ait puanın toplanmasıyla elde edilir, hangi sorunun hangi alt ölçeğe ait olduğu yukarıdaki tablolar kullanılarak anlaşılabilir ve tablolar yardımıyla alt ölçek puanları ve toplam puan elde edilir. Alt ölçek puanları 0-49, tüm ölçeğin puanı ise 0-196 arasında değişen değerlerde elde edilir. Yüksek değerler sağlıkla iilişkili yaşam kalitesinin yüksekliğini, düşük değerler azalmış sağlıkla ilişkili yaşam kalitesini ifade eder. Her alt ölçekte en fazla bir eksik madde bulunabilir, değerlendirme sırasında alt ölçeğin bireysel medianı boş bırakılan maddenin değeri yerine konularak hesaplama yapılır. Eğer bir alt ölçekte birden fazla soru yanıtlanmadıysa, bu soru kağıdını bilimsel değerlendirmeler için kullanmamanızı önerilir. Bireysel değerlendirmelerde cinsiyet göz önünde bulundurulmalıdır (genel olarak çölyak hastası kadınlarda sağlıkla ilişkili yaşam kalitesi çölyak hastası olan erkeklere göre daha düşük belirlenmektedir; Häuser et al. 2007). Müdahale çalışmaları gibi tekrarlı ölçüm yapılacak çalışmalarda (örn. bir tedavinin sağlıkla ilişkili yaşam kalitesi üzerine etkilerini tespit etmek için) analizlerdeki minimum anlamlı puan farkının toplamda ≥ 12 ve alt ölçelerde ≥ 3, olması klinik açıdan önemli fark olarak kabul edilmesi uygundur. Gruplar arası karşılaştırma yapılan analizlerde ise minimum ≥ ½ standart sapma değişiminin klinik açıdan önemli fark olarak değerlendirilmesi gerekmektedir.

Telif Hakları

CDQ’nun türkçe versiyonunun kullanımı için ilgili kişiden izin alınması ve ilgili makalenin kaynak gösterilmesi koşuluyla herhangi bir telif hakkı ödemesi olmaksızın klinik çalışmalarda kullanılması uygundur. İlgili kişilere, müelliften izin talep ettikten sonra, kullanım izni verilecektir (email üzerinden: aysegul.cakmak@hacettepe.edu.tr)
